# Evaluation of the association between vitamin D and lung cancer skin metastasis: A protocol for systematic review

**DOI:** 10.1097/MD.0000000000023281

**Published:** 2020-12-04

**Authors:** Dan Zhao, Tao Wang, Yu-feng Li, Jian-wei Huang

**Affiliations:** a Department of Dermatology, Second Affiliated Hospital of Mudanjiang Medical University; b Department of Chest Surgery, The Affiliated Hongqi Hospital of Mudanjiang Medical University, Mudanjiang, China.

**Keywords:** association, lung cancer, skin metastasis, vitamin D

## Abstract

**Background:**

This study aims to investigate the association between vitamin D (VD) and lung cancer skin metastasis (LCSM).

**Methods:**

The following databases will be retrieved from the beginning to the present of each database without language limitation: PUBMED, EMBASE, Cochrane Library, Web of Science, CBM, and CNKI. The reference lists of included trials and other sources will also be checked. Two researchers will independently undertake literature selection, data collection, and study quality evaluation. We will utilize a fixed or random-effect model to pool the data according to the heterogeneity test. The RevMan 5.3 software will be used to analyze the data and perform meta-analysis.

**Results:**

This study will summarize high quality study to explore the association between VD and LCSM.

**Conclusion:**

The findings of this study will help to judge whether there is association between VD and LCSM.

**Ethics and dissemination:**

No research ethical approval is required in this study, because it will only analyze published data. It is expected to disseminate through a peer-reviewed journal.

**Study registration:**

osf.io/ph2au.

## 1 Introduction

Lung cancer (LC) is one of the most common cancers around the world.^[1–4]^ It is also the leading cause of cancer-related mortality with about 32% and 25% in men and women, respectively.^[5–8]^ Other studies found that its survival is substantially influenced by different stages at diagnosis.^[9–12]^ It is reported that its 5-year survival ranges from 92% (earliest stage) to 0% (latest stage), respectively.^[7]^ Some patients with LC often have metastasis to other organs, such as lung cancer skin metastasis (LCSM).^[13–18]^ Previous studies have reported that vitamin D (VD) has association with LCSM.^[19–31]^ However, there are still inconsistent findings among them. In addition, no study has investigated this topic at evidence-based medicine level. Thus, this study will explore the association between VD and LCSM systematically and comprehensively.

## 2 Methods

### 2.1 Objective

This study protocol aims to investigate the association between VD and LCSM.

### 2.2 Study registration

This study has been registered on OSF (osf.io/ph2au). It is reported based on the guideline of Preferred Reporting Items for Systematic Reviews and Meta-Analysis Protocol statement.^[32]^

### 2.3 Inclusion criteria for study selection

#### 2.3.1 Type of studies

Case-controlled study (CCS) will be considered for inclusion in this study. Other types of studies will be excluded, such as animal study, case report, case series, and review.

#### 2.3.2 Type of participants

This study will include subjects who were diagnosed as LCSM or normal participants without restrictions to race, age, and sex. However, patients will be excluded if they had skin metastasis from other cancers, or skin cancer alone.

#### 2.3.3 Type of exposures

This study will test serum levels of VD in patients with LCSM.

#### 2.3.4 Type of controls

This study will examine serum levels of VD in normal healthy participants.

#### 2.3.5 Type of outcome measurements

Outcomes include levels of serum vitamin D, number of skin metastasis, disease progression rate, overall survival rate, mortality rate, and quality of life.

### 2.4 Search methods for the identification of studies

PubMed, EMBASE, Cochrane Library, Web of Science, CBM, and CNKI will be retrieved from the beginning to the present without language limitation. The search strategy sample for PubMed is demonstrated in Table 1. It will be modified by using other electronic databases. Additionally, the reference lists of included trials, conference papers related to the topic, and clinical trials registry will be searched to avoid missing potential studies.

**Table 1:** Detailed search strategy of Cochrane Library.

Number	Search terms
1	MeSH descriptor: (Lung Neoplasms) explode all trees
2	MeSH descriptor: (Skin Neoplasms) explode all trees
3	((Neoplasm, Lung^∗^) or (Lung Neoplasm^∗^) or (Neoplasm, Pulmonary ^∗^) or (Pulmonary Neoplasm^∗^) or (Lung Cancer^∗^) or (Cancer, Lung^∗^) or (Pulmonary Cancer^∗^) or (Cancer, Pulmonary^∗^) or (Cancer of Lung ^∗^) or (Lung Carcinoma, Non-Small-Cell^∗^) or (Non Small Cell Lung Carcinoma^∗^) or (Non-Small Cell Lung Cancer^∗^) or (Skin Cancer^∗^) or (Cutaneous Cancer^∗^) or (Cutaneous Malignancy^∗^) or (Skin Neoplasms^∗^) or (Skin Metastasis^∗^)):ti, ab, kw
4	Or 1–3
5	MeSH descriptor: (Vitamin D) explode all trees
6	((Vitamin D^∗^) or (25-hydroxuvitain D^∗^) or (25(OH) vitamin D^∗^)):ti, ab, kw
7	Or 5–6
8	MeSH descriptor: (Case-control Studies) explode all trees
9	((Case-control Study^∗^) or (Case-referent Study^∗^) or (Case-controlled Study^∗^) or (Observational Study^∗^) or (Cohort Study^∗^) or (Studies^∗^)):ti, ab, kw
10	Or 8–9
11	4 and 7 and 10

### 2.5 Data collection and analysis

#### 2.5.1 Selection of studies

All searched literature will be imported into Endnote X9.0 software, and duplicates will be excluded. Then, 2 researchers will independently scan the titles, abstracts, and full texts of potential citations to select included studies. The reasons for all excluded studies will be recorded in a table. Any confusion will be cleared up by consulting a third researcher. A flow chart will be utilized to present study selection process.

#### 2.5.2 Data collection and management

Two researchers will independently extract necessary information from included studies. It consists of title, first author, year of publication, journal, location, patient demographics, types of exposures, outcome measurements, results/findings, limitations, conflict of interest, and funding information. We will consult a third researcher if there are divisions between 2 authors.

#### 2.5.3 Study quality assessment

The study quality of all included CCS will be evaluated using The Newcastle-Ottawa Scale.^[33,34]^ All included studies will be graded as high, moderate, and low risk according to the criteria of this tool. Any discrepancy will be settled down by consensus between 2 researchers and an additional researcher.

#### 2.5.4 Dealing with missing data

To obtain unclear or missing data, we will contact first or corresponding author to obtain it by email or telephone. If that fails, we will analyze the existing data using intention to treat analysis.

#### 2.5.5 Assessment of heterogeneity

*I*^2^ test will be used to assess statistical heterogeneity. *I*^2^ ≤ 50% is considered as low heterogeneity, and we will employ a fixed-effect model to pool the data. *I*^2^ > 50% is considered as substantial heterogeneity, and we will use a random-effect to synthesize the data. Meanwhile, sensitivity or subgroup analysis will be performed to investigate the possible causes of heterogeneity.

#### 2.5.6 Assessment of reporting biases

If sufficient number of included studies (over 10 studies) is entered, we will examine reporting bias using funnel plot and Egger regression test.^[35]^

#### 2.5.7 Statistical analysis

RevMan 5.3 software will be employed for data synthesis. For dichotomous outcome data, risk ratio or odds ratio and 95% confidence intervals (CIs) will be used to calculate treatment effect. Mean difference (MD) or standardized MD and 95% CIs will be utilized to estimate treatment effect for continuous data. Whenever possible, we will carry out a meta-analysis if sufficient data are extracted on the same outcome with ample similarity in study information, patient demographics, details of exposures, and outcomes. If outcome data cannot be synthesized, and a meta-analysis cannot be performed, we will undertake descriptive analysis.

#### 2.5.8 Subgroup analysis

If it is necessary, we will carry out a subgroup analysis based on different types of study information, exposures, and outcomes.

#### 2.5.9 Sensitivity analysis

We will conduct a sensitivity analysis to check the stability of combined outcome results according to sample size, methodological quality, and missing data.

## 3 Discussion

This study will firstly investigate the association between VD and LCSM. We will systematically and comprehensively search literature resources, including electronic databases and grey literature sources to avoid missing potential studies. Two researchers will independently conduct study selection, data extraction, and study quality assessment, respectively. Any confusion between 2 researchers will be solved by a third researcher through discussion, and a consensus will be reached after discussion. If it is possible, we will pool the data and carry out meta-analysis. If it is not possible to synthesize the data, we will report study findings by narrative description. The findings of this study will provide evidence to support the association between VD and LCSM.

## Author contributions

**Conceptualization:** Dan Zhao, Tao Wang, Yu-feng Li, Jian-wei Huang.

**Data curation:** Dan Zhao, Jian-wei Huang.

**Formal analysis:** Dan Zhao, Tao Wang, Yu-feng Li, Jian-wei Huang.

**Investigation:** Jian-wei Huang.

**Methodology:** Dan Zhao, Tao Wang, Yu-feng Li.

**Project administration:** Jian-wei Huang.

**Resources:** Dan Zhao, Tao Wang.

**Software:** Dan Zhao, Tao Wang, Yu-feng Li.

**Supervision:** Jian-wei Huang.

**Validation:** Dan Zhao, Tao Wang, Yu-feng Li, Jian-wei Huang.

**Visualization:** Dan Zhao, Tao Wang, Jian-wei Huang.

**Writing – original draft:** Dan Zhao, Yu-feng Li, Jian-wei Huang.

**Writing – review & editing:** Dan Zhao, Tao Wang, Yu-feng Li, Jian-wei Huang.
